# CILP, a Putative Gene Associated With Immune Infiltration in Breast Cancer Brain Metastases

**DOI:** 10.3389/fgene.2022.862264

**Published:** 2022-05-30

**Authors:** Xiaolin Sun, Ning Yang, Xingguo Zhou, Honghai Dai, Qiang Li, Alei Feng, Gongwen Xu, Yingchao Liu, Linzong Xu, Zhanyu Zhang, Zhe Yang, Xiaomei Li

**Affiliations:** ^1^ Tumor Research and Therapy Center, Shandong Provincial Hospital Affiliated to Shandong First Medical University, Jinan, China; ^2^ Tumor Research and Therapy Center, Shandong Provincial Hospital, Cheeloo College of Medicine, Shandong University, Jinan, China; ^3^ Department of Gastrointestinal Surgery, The Second Hospital, Cheeloo College of Medicine, Shandong University, Jinan, China; ^4^ Business School, Shandong Jianzhu University, Jinan, China; ^5^ Department of Neurosurgery, Shandong Provincial Hospital Affiliated to Shandong First Medical University, Jinan, China

**Keywords:** breast cancer, brain metastases, CILP, prognosis, immune infiltration

## Abstract

Breast cancer (BC) is the second leading cause of brain metastases (BM), with high morbidity and mortality. The aim of our study was to explore the effect of the cartilage intermediate layer protein (CILP) on breast cancer brain metastases (BCBM). Using a weighted gene coexpression network analysis (WGCNA) in GSE100534 and GSE125989 datasets, we found that the yellow module was closely related to the occurrence of BCBM, and CILP was a hub gene in the yellow module. Low CILP expression was associated with a poor prognosis, and it was an independent prognostic factor for stage III–IV BC determined using Cox regression analysis. A nomogram model including CILP expression was established to predict the 5-, 7-, and 10-year overall survival (OS) probabilities of stage III–IV BC patients. We found that CILP mRNA expression was downregulated in BCBM through GSE100534, GSE125989, and GSE43837 datasets. In addition, we found that CILP mRNA expression was negatively correlated with vascular endothelial growth factor A (VEGFA), which is involved in regulating the development of BM. UALCAN analysis showed that CILP expression was downregulated in HER2-positive (HER2+) and triple-negative breast cancer (TNBC), which are more prone to BM. The vitro experiments demonstrated that CILP significantly inhibited BC cell proliferation and metastasis. Western blot (WB) results further showed that the mesenchymal protein marker vimentin was significantly downregulated following CILP overexpression, suggesting that CILP could participate in migration through epithelial–mesenchymal transition (EMT). A comparison of CILP expression using immunohistochemistry in BC and BCBM showed that CILP was significantly downregulated in BCBM. In addition, gene set variation analysis (GSVA) revealed that CILP was associated with the T-cell receptor signaling pathway in BCBM and BC, indicating that CILP may be involved in BCBM through immune effects. BCBM showed lower immune infiltration than BC. Moreover, CILP expression was positively correlated with HLA-II, T helper cells (CD4^+^ T cells), and Type II IFN Response in BCBM**.** Collectively, our study indicates that CILP is associated with immune infiltration and may be a putative gene involved in BCBM. CILP offers new insights into the pathogenesis of BCBM, which will facilitate the development of novel targets for BCBM patients.

## 1 Introduction

Among all solid tumors, breast cancer (BC) is the second most common cause of brain metastases (BM) (Nayak et al., 2012; Witzel et al., 2016; Pellerino et al., 2020). Approximately 15–30% of patients with BC eventually develop BM with high morbidity and mortality (Nayak et al., 2012; Tabouret et al., 2012). BM occurs in 30–55% of patients with HER2+ metastatic BC, and up to half die from intracranial progression, whereas the median survival rate is only six months in triple-negative BC (TNBC) with BM (Lin et al., 2008; Dawood et al., 2009). Unfortunately, effective treatment is not available because the central nervous system is traditionally considered an immune-privileged site due to the blood–brain barrier (BBB) (Witzel et al., 2016; Kotecki et al., 2018). As a result, the identification of genetic and epigenetic alterations is essential for developing the BM-targeted therapies (Pedrosa et al., 2018).

Although research on the mechanism involved in BCBM has been ceaseless, yet it remains largely unclear. Bos et al. (2009) showed that ST6GALNAC5 acts as a specific mediator of BCBM by enhancing its adhesion to the brain endothelial cells and facilitating the crossing of the BBB by BC cells. Highly sialylated N-glycans, that were upregulated in the brain-seeking cell line 231BR, are likely to play a crucial role in BCBM, as evaluated using integrated transcriptomics, glycomics, and proteomics (Peng et al., 2019). GABA_A_ receptor alpha3 (GABRA3), normally exclusively expressed in the adult brain, is inversely correlated with BC survival and promotes BC cell invasion and BM by activating the AKT pathway (Gumireddy et al., 2016). Recently, it was also reported that YTHDF3 could enhance the translation of m6A-enriched transcripts for ST6GALNAC5, EGFR, and GJA1, thus promoting BCBM (Chang et al., 2020). To date, studies on the mechanism of BCBM are limited. Hence, our study is aimed to explore the new potential mechanism to guide the treatment of BCBM.

The tumor microenvironment (TME), composed of diverse immune cells, tumor cells, and cytokines, has both adverse and beneficial impacts on tumorigenesis (Kim and Bae, 2016). There is growing evidence that an ineffective immune response influences the behavior of BC, suggesting that it is an immunogenic cancer type (Duchnowska et al., 2016). BC is placed in the category of cold immunity (Mortezaee, 2021). Cold cancers have a noninflamed TME, which is prone to evade antitumor immune cells because tumoral cells lack sufficient neoantigens. Low to moderate T-cell recruitment has been observed in the BM of patients with BC (Friebel et al., 2020). A recent study showed that CD4^+^ T cells from the BM show an anergic, hyporesponsive phenotype, while CD8^+^T cells display exhaustion characteristics (Klemm et al., 2020). Immunosuppressive microenvironment in BM prevents T-cell initiation and infiltration, which is one of the causes of T-cell dysfunction. Transforming growth factor -beta (TGF-β) is an immunosuppressive factor that affects the differentiation and function of T cells and prevents T cells from infiltrating into tumors, thus promoting immune escape of tumor cells. Hence, it is important to understand the mechanisms of immune regulation in BCBM.

Herein, we used a series of bioinformatics tools, such as WGCNA, to identify whether CILP inhibits BCBM. Next, we proved that CILP functionally inhibited the proliferation and metastasis of BC cells *in vitro*. In addition, we used the bioinformatics analysis, including the microenvironment cell population (MCP)-counter, Estimation of Stromal and Immune cells in Malignant Tumor tissues using Expression data (ESTIMATE), and single-sample gene set enrichment analysis (ssGSEA), to demonstrate the relationship between immune microenvironment of BCBM and CILP expression.

## 2 Materials and Methods

### 2.1 Clinical Specimens and Ethics Statement

Sixteen paraffin-embedded BC tissues, including nine cases of primary BC tissues and seven cases of BCBM tissues, were collected from Shandong Provincial Hospital. The study was approved by the Biomedical Ethics Committee of Shandong Provincial Hospital.

### 2.2 Data Acquisition and Processing

The GSE43837, GSE100534, and GSE125989 datasets were obtained from the NCBI Gene Expression Omnibus (GEO) (McMullin et al., 2014; Schulten et al., 2017; Iwamoto et al., 2019). GSE43837 contains RNA sequences of 19 HER2+ BCBM and 19 non-BM breast cancers samples. GSE125989 consists of 32 samples with matched primary BC and BCBM from 16 patients. From GSE100534, we obtained 19 samples, including 3 BCBM samples and 16 BC samples. The R packages of “GEOquery” and “oligo” were used to access and process the data, using the Robust Multichip Average (RMA) algorithm, followed by normalization of the raw data (.CEL files) and matrix construction. The R packages of “hugene10sttranscriptcluster” (GPL6244) and “hgu133a2” (GPL571) were used to match the probe to their gene symbols, and the probes matching several genes were removed. For genes matched by multiple probes, we selected the probe with the highest average expression in the samples. We combined the GSE100534 and GSE125989 datasets using a Perl script, then used the “sva” package (under the R environment, version 3.6.3) to preprocess and remove the batch effect and eventually obtained a merged dataset. Microarray annotation information in GPL1352 was used to match the probes with the corresponding genes of GSE43837, and the median expression value of genes matched with multiple probes was calculated. The fragments per kilobase million (FPKM) values of the RNA-seq data were downloaded from the Cancer Genome Atlas (TCGA) database and obtained 1,208 samples (normal: 112 and tumor: 1,096). The samples lacking clinical information were excluded.

### 2.3 Analysis of Differentially Expressed Genes

We performed a differential analysis comparing BC tissues and BCBM tissues using the GEO datasets. Differentially Expressed Genes (DEGs) were displayed in a heatmap and histograms, screened, and matrixes were constructed using the “limma” package in RStudio using |log2FoldChange (FC)|≥ 1 and adjusted *p* value < the 0.05 as cutoff values.

### 2.4 Construction of Weighted Gene Coexpression Network

The data used were from the merging datasets GSE100534 and GSE125989, as described in Section 2.2. We chose all the 11,786 genes for weighted gene coexpression network analysis (WGCNA), and the power parameter was precalculated using the pickSoftThreshold function (Langfelder and Horvath, 2008). Calculating the exponentials for several powers in a scale-free topology fit makes it possible to obtain an appropriate soft threshold power for network construction. After choosing the appropriate soft thresholding power, adjacency was transformed into a topological overlap matrix (TOM), which measured the network connectivity of genes (Botia et al., 2017). To classify genes with similar expression profile into gene modules, average linkage hierarchical clustering was performed according to the TOM-based dissimilarity measure with a minimum module size of 30 for the gene dendrogram (Rahmani et al., 2016). To further analyze the module, we merged the highly similar modules with a dissimilarity of <0.25 by clustering module eigengenes (MEs). Next, we calculated the correlation between MEs and clinical features of BCBM. As a representative of all the genes in each module, MEs were defined as the first principal component of each gene module. Gene significance (GS) was defined as the log10 transformation of the *p* value (GS = log_10_ *p*) in the linear regression between the gene expression of the module and clinical features. Module significance was defined as the mean GS of all the genes in the module. Visualization of the eigengene network was displayed using Cytoscape 3.8.0.

### 2.5 Biological Function and Pathway Enrichment Analysis

In gene networks that conform to scale-free distributions, genes with similar expression pattern could be synergistically regulated, pathway shared, or functionally related. We selected the module of the most relevant clinical characteristics, and then performed gene ontology (GO) and kyoto encyclopedia of genes and genomes (KEGG) pathway analysis *via* the “clusterprofiler” package in RStudio software (Yu et al., 2012). GSVA was applied to explore the difference in biological pathway, using the R package GSVA. The gene sets of “c2.cp.kegg.v7.4.symbols” was downloaded from the MSigDB for GSVA.

### 2.6 Kaplan–Meier Plotter Analysis and CILP Expression in Breast Cancer

The correlation between CILP expression and survival in BC was analyzed using the Kaplan–Meier plotter. Hazard ratio (HR) with 95% confidence interval (CI) and log-rank *p* value were computed. The LinkedOmics database, which is used to analyze 32 TCGA cancer-related multidimensional datasets, is a web-based platform. The data types include microRNAs, single nucleotide polymorphisms, methylation status, gene mutations, and clinical data (Vasaikar et al., 2018). First, a nonparametric test was used to analyze the clinical data of the invasive breast carcinoma cohort in TCGA, and the transcriptional variation in CILP expression in different clinical stages (Kruskal–Wallis test) and the M stage (Wilcox test) were evaluated using this platform. Next, we applied UALCAN to analyze the transcriptional levels of *CILP* in BC (Chandrashekar et al., 2017).

### 2.7 Levels of Immune Infiltration in Breast Cancer and Breast Cancer Brain Metastases

We used the MCP-counter and ESTIMATE R packages to evaluate tumor purity, immune score, and stromal score for the patients with tumors. The levels of 29 immune- and tumor-related markers in each sample were quantified using ssGSEA. We also assessed 22 types of immune cells utilizing the “Cell type Identification by Estimating Relative Subsets of RNA Transcripts” (CIBERSORT) algorithm. Only those samples with a CIBERSORT output of *p* < 0.05 were considered for further analysis. Significant differences in the proportion of immune infiltrating between BC and Breast Cancer Brain Metastases (BCBM) tissues were determined by the Wilcoxon rank-sum test. Furthermore, infiltration of immune cells in the two groups with a low and high CILP expression was analyzed.

### 2.8 Cell Culture, Transient Transfection, and Quantitative Real-Time PCR

BC cell lines, including MDA-MB-231, MDA-MB-468, and MCF-7, were obtained from the American Type Culture Collection (ATCC, Manassas, VA, United States). The cells were cultured in Leibovitz’s L15 medium (MDA-MB-231 and MDA-MB-468) or Dulbecco’s modified Eagle’s medium (DMEM, MCF-7) supplemented with 10% fetal bovine serum (FBS, Gibco) at 37°C under 5% CO_2_ routinely. Lipofectamine 2000 (Invitrogen, Carlsbad, CA, United States) was used for transfecting the pCMV3-CILP-FLAG plasmid (HG29937-CF, Sino Biological) into BC cells according to the manufacturer’s instructions. All the constructs were confirmed by DNA sequencing. The total RNA was extracted with TRIzol reagent according to the manufacturer’s instruction (Invitrogen). A LightCycler (ABI PRISM® 7000) and SYBR RT-PCR kit (Takara) were used for the Quantitative Real-Time PCR (RT-qPCR) analysis. The specific primer sequences shown as follows: β-ACTIN, Forward(F): 5′-CAT​GTA​CGT​TGC​TAT​CCA​GGC-3′, Reverse(R): 5′- CTC​CTT​AAT​GTC​ACG​CAC​GAT-3′; CILP, Forward(F): 5′- GGC​AAC​CTG​GAG​ATT​CGT​GA-3′, Reverse (R): 5′- CAC​CTT​AAC​AAA​GCA​CCG​CC -3′. In each sample, the data are normalized to β-actin expression.

### 2.9 Colony Formation and CCK-8 Assays

For the CCK-8 assay, 3,000 cells were inoculated into each well of 96-well plate after transfection with pCMV-FLAG and pCMV3-CILP-FLAG plasmids for 24 h. The cell proliferation level was determined using the Cell Counting kit-8 (CCK-8) (Dojindo), according to the manufacturer’s instructions. Next, 10 µl CCK-8 working fluid was added to each well at 24, 48, 72, 96, and 120 h after transfection and cultured in a 5% CO_2_ incubator at 37°C for 2 h. Finally, the absorbance was measured at 450 nm using an Infinite M200 PRO microplate reader (Tecan). For the colony formation assay, 800 cells were seeded into each well of the 6-well plate (Costar, Corning, NY, United States), cultured for 10–15 days, and then, stained with crystal violet (Solarbio, Beijing, China).

### 2.10 Wound Healing Assay and Transwell Assay

The migration and invasion of BC cells were determined using wound healing and transwell assays after the transfection with the CILP plasmid. For the wound healing assay, the transfected cells were plated into 6-well plate at a density of 1,000,000 cells per well. Next, a sterile tip (20 µl) was used to scratch the cell monolayer. The detached cells were removed with phosphate-buffered saline and cultured in a serum-free L15 medium. Finally, a microscope was used to observe the scratch wound after 48 h. The transwell assay was performed according to the study protocol (Zhang et al., 2019).

### 2.11 Western Blotting and Immunohistochemistry Staining

WB and Immunohistochemistry (IHC) were performed as described in the previous studies (Huai et al., 2016; Yang et al., 2021). Anti-Vimentin 5741 was from Cell Signaling Technology. Anti–CILP (ab192881) was from Abcam. Anti–CILP (HPA003195) was from Atlas. Anti-Flag (F1804) was from Sigma. Anti–β-actin (sc-81178) and horseradish peroxidase-conjugated secondary antibodies were from Santa Cruz Biotechnology.

### 2.12 Statistical Analysis

All the statistical analyses were performed using GraphPad, R 3.6.3, R 4.1.1, and Perl 5 (v5.30.0). Survival analysis was performed using the “survminer” and “survival” packages in RStudio software. We then used the surv_cutpoint function to obtain the best cutoff by dividing the gene into high and low expression groups for clinical analysis. Univariate and multivariate Cox regression analyses were performed to establish Cox proportional hazard regression and nomogram models. The features with *p* value < 0.05 in univariate analysis were selected for multivariate analysis. We drew 5-, 7-, and 10-year receiver operating characteristic (ROC) curves and the calibration curves to evaluate the model’s accuracy and discrimination using the “timeROC” R package (Blanche et al., 2013). We used the *t*-test of equal variance hypothesis and Chi-square test. All the experimental data are presented as the mean ± standard deviation. The strength of Spearman’s correlation was determined using the following guide for the absolute value: 0.00–0.19, “very weak”; 0.20–0.39, “weak”; 0.40–0.59, “moderate”; 0.60–0.79, “strong”; and 0.80–1.0, “very strong”. Statistical significance was described as follows: *ns*, not significant; *p < 0.05; **p < 0.01; ***p < 0.001; and ****p < 0.0001.

## 3 Results

### 3.1 Genes in the Yellow Module May Mediate Breast Cancer Brain Metastases

**FIGURE 1:**
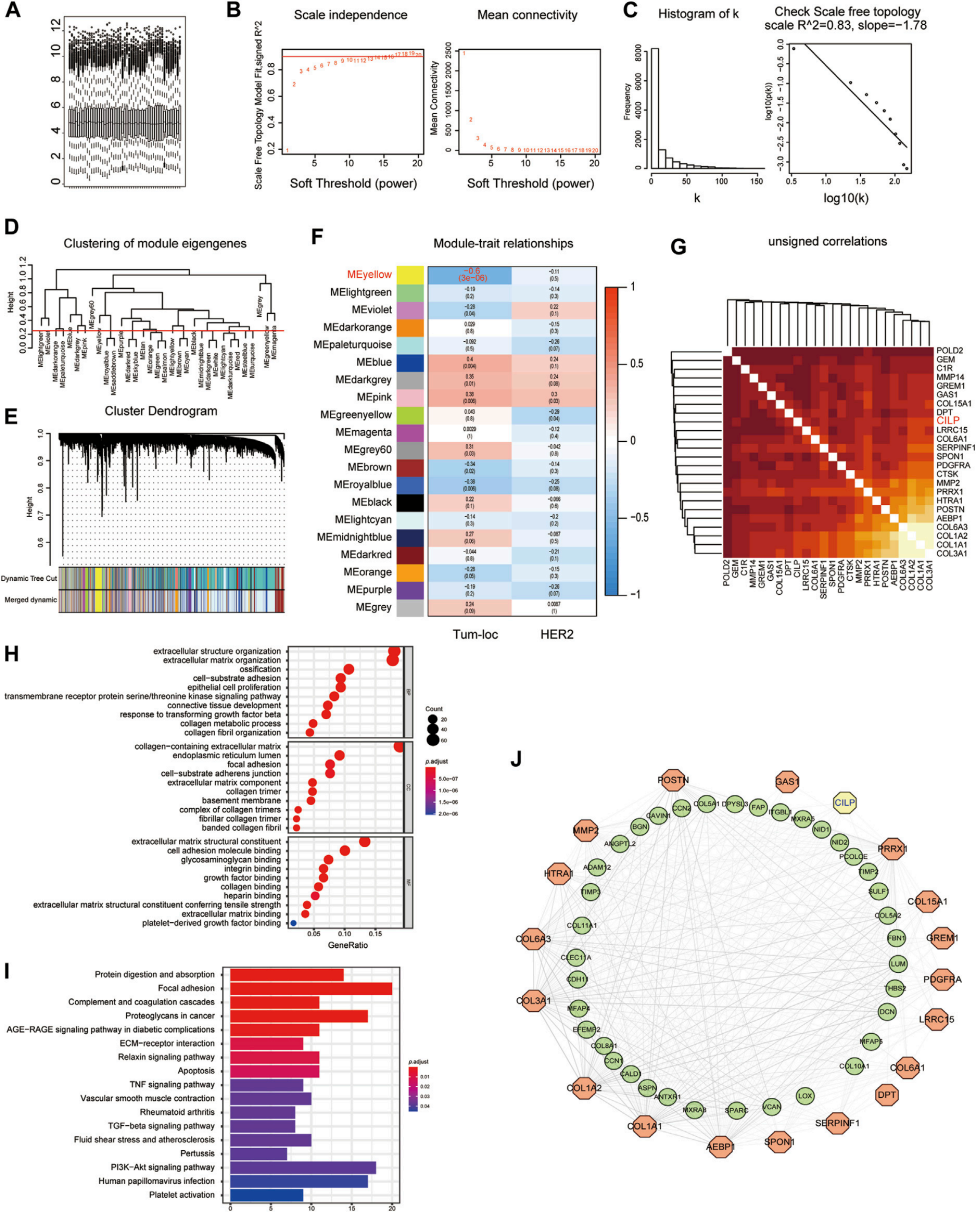
Yellow module is identified as the key module associated with BCBM using WGCNA. **(A)** Box plot of gene expression of the merged datasets of GSE125989 and GSE100534 after normalization using the “sva” package. **(B)** Scale-free fit index and the mean connectivity analysis for various soft-thresholding powers. The panel on the left shows the relationship between the soft-threshold and the scale-free R2. The right panel shows the relationship between the soft-threshold and average connectivity. **(C)** The soft-thresholding power (*β* = 8) in the WGCNA was determined based on a scale-free R_2_ (R_2_ = 0.83). **(D)** Cluster dendrogram of MEs. Implementing clustering of MEs by merging the highly similar modules with the dissimilarity of <0.25. **(E)** Dendrogram of all the expressed genes clustered based on different metrics. Each branch in the figure denotes one gene, and each color below represents one coexpression module. **(F)** Heatmap showing the correlation between the clinical traits and MEs. The yellow module containing 399 genes has the highest negative correlation with the BCBM phenotype. Each cell contains the correlation and *p* value. The correlation coefficient of each cell indicates the correlation between the gene module and clinical traits, which reduces in size from red to blue. Tum-loc represents the location of the tumor, including BC and BCBM tissues. HER2 represents the positive or negative status. **(G)** Heatmap showing 24 candidate hub genes for the yellow module in **(F)**. **(H)** Enriched GO terms in cellular component (CC), biological process (BP), and molecular function (MF) categories for the yellow module in **(F)**. Different sizes denote the number of genes, while different colors denote different significance. **(I)** KEGG pathways were analyzed for all the genes of the yellow module in **(F)**. The length of the column indicates the enrichment score, while the colors represent enrichment significance. **(J)** Protein–protein interaction (PPI) network for all the genes in the yellow module from **(F)** obtained using Cytoscape, consists of 56 nodes and 366 edges according to the weight of the edge (≥0.07).

Considering the poor prognosis of patients with BM, we performed WGCNA to search for the hub genes related to BM. WGCNA is a systematic biological approach used to analyze the expression pattern of multiple genes in different samples, to form clusters or modules containing genes with the same expression pattern (Beckerman et al., 2017). If certain genes are located in the same module, they are likely to possess the same biological functions (Rosen et al., 2011; Tang et al., 2018). The raw data from the GSE100534 and GSE125989 datasets were preprocessed identically using RStudio for background correction and normalization (Figure 1A). In this study, we selected the power of *β* = 8 (scale-free R^2^ = 0.83) as the soft thresholding to achieve a scale-free network (Figures 1B,C). As a result, 20 gene coexpression modules were identified using a merged dynamic tree with a cutoff height of 0.25 (Figures 1D,E). The yellow module showed the highest correlation with the BM phenotype of breast carcinoma (R^2^ = −0.6, *p* < 0.001), indicating that genes in the yellow module (399 genes) may play an important role in invasion and metastasis (Figure 1F). Furthermore, 24 genes were identified as the candidate hub genes for the yellow module (Figure 1G). Therefore, the yellow module was identified as the critical module associated with BCBM.

GO and KEGG enrichment analyses were performed, using the ClusterProfiler R package, to explore biological processes and pathways. The “biological process” category showed that the genes in the yellow module were enriched in the processes, such as extracellular matrix organization, cell-substrate adhesion, epithelial cell proliferation, and response to TGF-beta (Figure 1H). Furthermore, KEGG analysis revealed that focal adhesion, ECM–receptor interaction, TNF signaling pathway, apoptosis, TGF-beta signaling pathway, and PI3K-AKT signaling pathway were associated with these genes (Figure 1I). Moreover, we constructed a network of protein–protein interactions (PPIs) for all the genes in the yellow module using Cytoscape, which consisted of 56 nodes and 366 edges, according to the weight of the edge (≥0.07) (Figure 1J). In summary, these results indicate that genes in the yellow module may mediate BCBM.

### 3.2 CILP, a Yellow Module Gene, Is Downregulated in Invasive Breast Cancer

**FIGURE 2:**
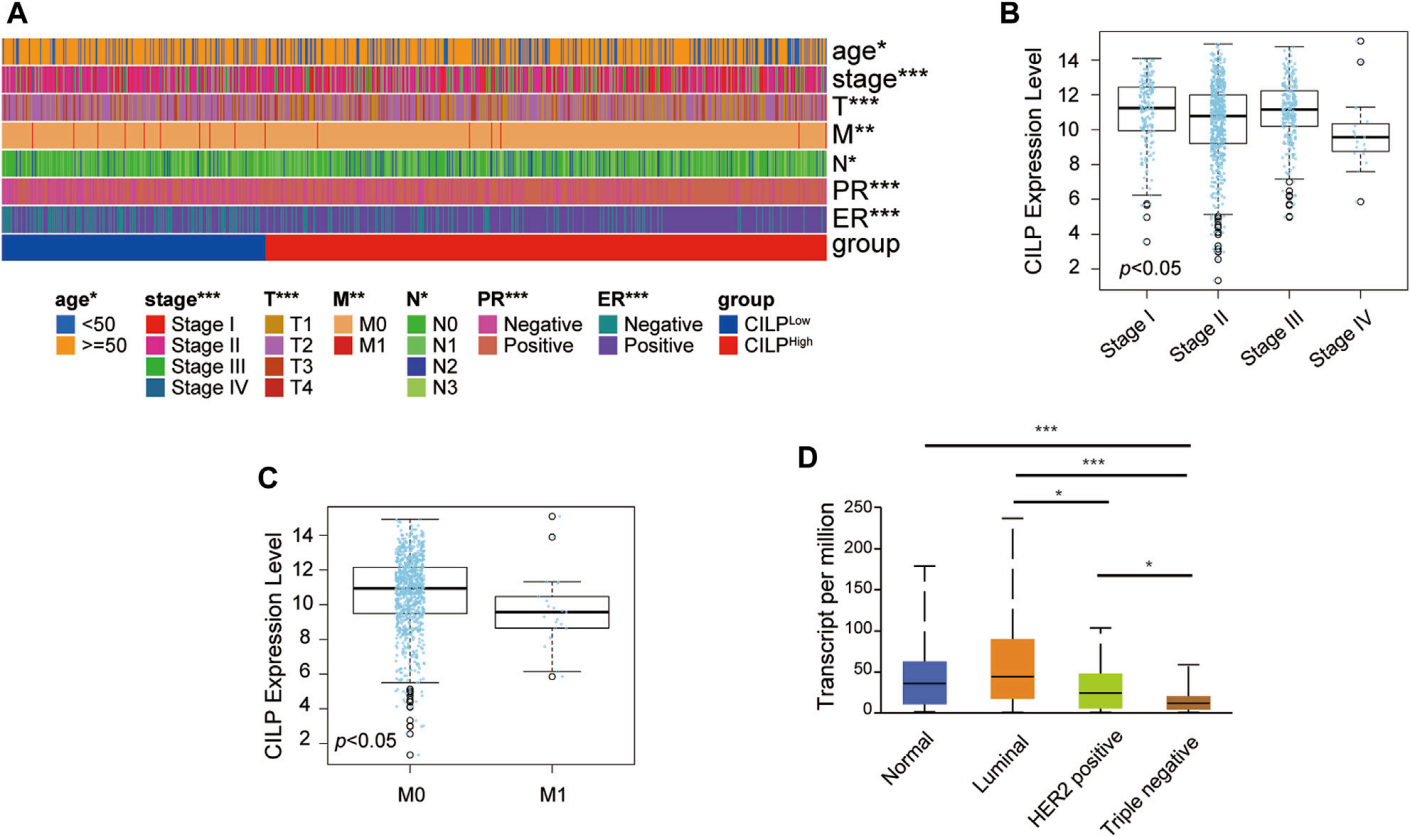
CILP expression in patients with invasive BC. **(A)** Heatmap for the clinicopathological features of patients with BC having high or low CILP expression. **(B,C)** Results from LinkedOmics database show the RNA expression level of CILP gene between different pathological stages **(B)** and the M stage **(C)** of patients with BC. **(D)** Results from UALCAN database show the association of CILP transcripts with intrinsic subclasses of BC. The *p* value of the data **(A)** is calculated by Wilcoxon rank-sum test. **(A)** TCGA cohort. *p < 0.05, **p < 0.01, and ***p < 0.001.

As shown in Figure 1G, CILP is the hub gene associated with the occurrence of BCBM. Therefore, the role of CILP in invasive BC requires further study. In the TCGA cohort, CILP displayed a differential expression pattern based on different clinical factors, including age, clinical stage, tumor size, lymph node status, metastasis, estrogen receptor (ER) status, and progesterone receptor (PR) status (Figure 2A). The LinkedOmics results showed that CILP mRNA levels were reduced in patients with stage IV and M1 (Figures 2B,C), suggesting that CILP may affect the prognosis of advanced BC. As shown in Figure 2D, the mRNA expression of CILP is different across the normal, luminal, HER2+, and triple-negative intrinsic subclasses of BC. In particular, HER2+ and triple-negative intrinsic subclasses are most prone to BM. Furthermore, the mRNA expression of *CILP* was significantly lower in HER2+ and TNBC than that in the luminal subclass. Overall, these results showed that low expression of CILP is implicated in metastasis.

### 3.3 CILP Affects the Prognosis of Stage III–IV Breast Cancer

**FIGURE 3:**
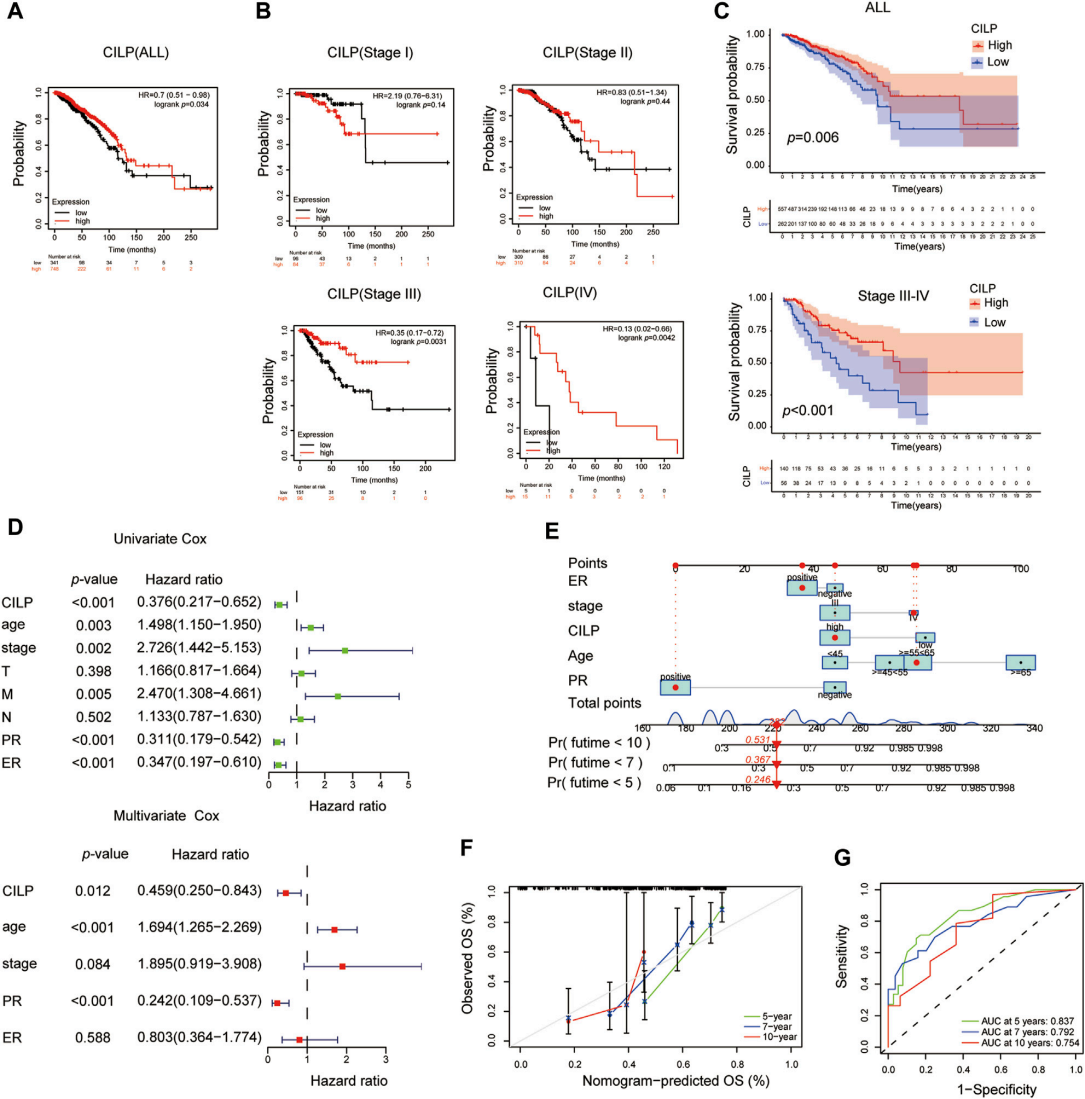
CILP affects the prognosis of patients with stage III-IV BC. **(A,B)** Kaplan–Meier survival analyses for patients with BC expressing high or low mRNA levels of *CILP* obtained using the Kaplan–Meier plotter database. **(C)** OS rate of the high-CILP expression group was better than that of the low-CILP expression group in BC, especially in patients with stage III-IV. **(D)** Forest maps of the univariate and multivariate Cox regression analysis in patients with stage III-IV BC. **(E)** Personalized scoring nomogram predicts 5-, 7-, and 10-year OS probability in patients with stage III-IV BC, and the arrow shows an example. **(F)** Calibration curves of 5-, 7-, and 10-year OS prediction in patients with stage III/IV BC. **(G)** ROC curve based on the nomogram exhibits the ability to predict 5-, 7-, and 10-year OS in patients with stage III-IV BC. Data **(C–G)**: TCGA cohort.

The Kaplan–Meier plotter database was used to evaluate the prognostic value of CILP in BC. Interestingly, a poor prognosis in BC was correlated with a lower CILP expression (Figure 3A). However, based on the RNA-seq data, CILP expression had no effect on survival in patients with early BC (stage I–II), and showed a worse overall survival (OS) in those with advanced BC (stage III–IV) (Figure 3B). To validate the abovementioned results, patients in the TCGA cohort were assigned into two groups based on the high and low CILP expression using the cutoff values obtained with the “survminer” package, and the survival analyses were conducted. Low expression of CILP resulted in markedly worse OS in patients with stage III-IV BC (Figure 3C). Further analysis showed that CILP expression was predictive of survival using univariate Cox regression analysis in the TCGA cohort. The factors (*p* < 0.05 in univariate Cox regression analysis) were then examined for the multivariate Cox’ regression analysis. CILP expression was considered to be an independent prognostic factor in patients with stage III–IV BC (Figure 3D). Based on the Cox’ regression model, a personalized scoring nomogram was established to predict the 5-, 7-, and 10-year OS probability of patients with BC using the five parameters (Figure 3E). The calibration curves of the OS prediction were close to the ideal model (45-degree line) (Figure 3F). As expected, the average area under the curve (AUC) values of 5-, 7-, and 10-year prognosis predictions reached 0.837, 0.792, and 0.754, respectively, which demonstrated that the nomogram exhibited a powerful capacity for the survival prediction (Figure 3G). Therefore, an integrated prognostic model could accurately predict OS in patients with stage III–IV BC. In conclusion, these results show that CILP is an independent prognostic factor in patients with stage III–IV BC.

### 3.4 CILP Inhibits the Proliferation, Migration, and Invasion of Breast Cancer Cells *In Vitro*

**FIGURE 4:**
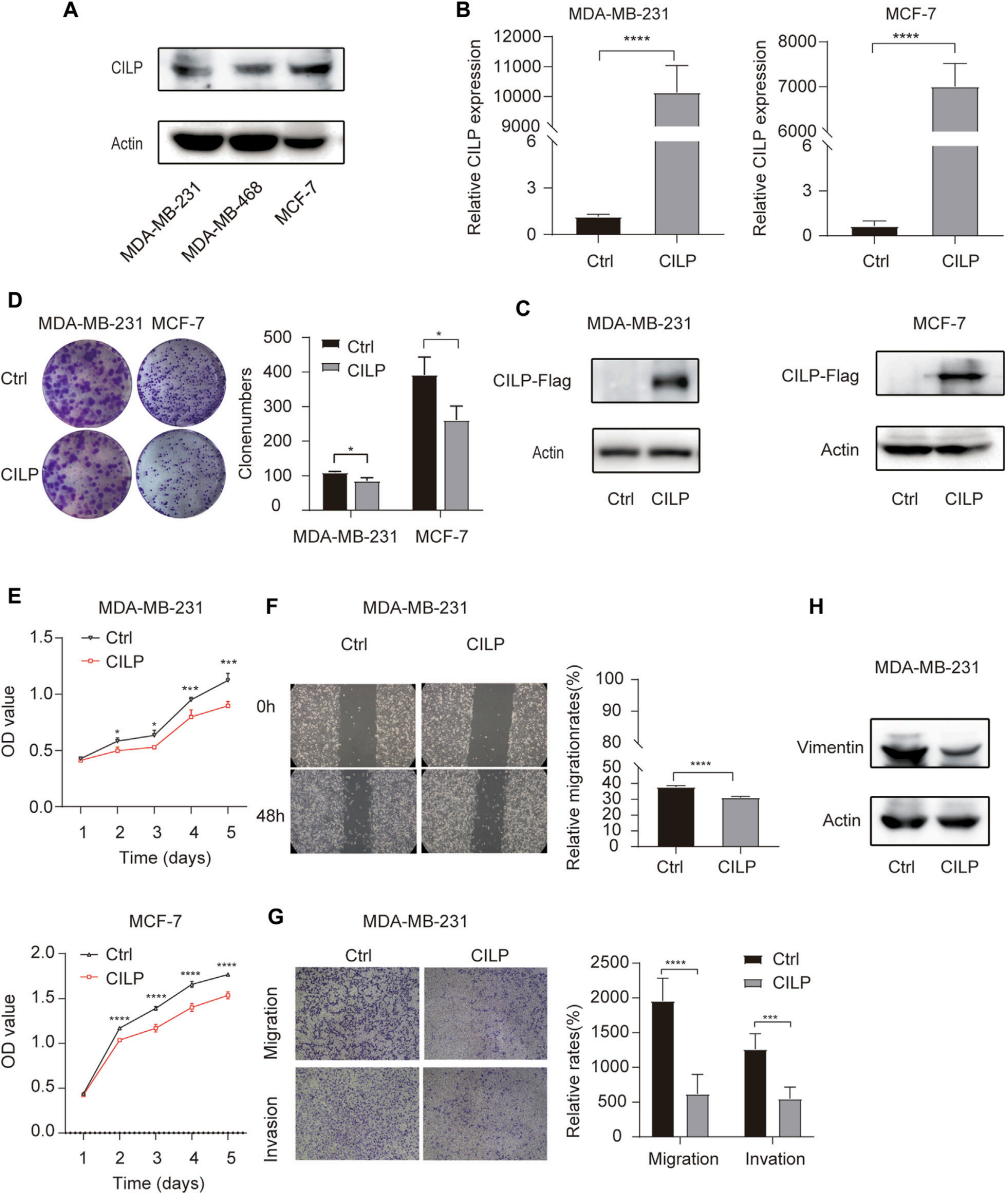
CILP inhibits the BC cell proliferation and metastases *in vitro*. **(A)** Expression of CILP protein in MDA-MB-231, MDA-MB-468, and MCF-7 cells was analyzed using WB. **(B,C)** RT-qPCR and WB analysis of CILP in MDA-MB-231 and MCF-7 cells transfected with CILP plasmid. **(D)** Representative images (the left side) and quantification (the right side) of control (Ctrl)- or CILP expression plasmid-transfected MDA-MB-231 and MCF-7 cells analyzed in the colony formation experiments. **(E)** Quantification of cell proliferation levels in the MDA-MB-231 and MCF-7 cells analyzed using the CCK-8 assay. **(F)** Representative images (the left side) and quantification (the right side) of control (Ctrl)- or CILP expression plasmid-transfected MDA-MB-231 were analyzed using the wound healing experiment. **(G)** Representative images (the left side) and quantification (the right side) of control (Ctrl)- or CILP expression plasmid-transfected MDA-MB-231, which were analyzed using the transwell assay. **(H)** WB analysis of a mesenchymal marker (vimentin) in control (Ctrl)- or CILP expression plasmid-transfected MDA-MB-231 cells. All the data are expressed as the mean ± S.E. (*n* = 3, *p < 0.05, **p < 0.01, ***p < 0.001, and ****p < 0.0001, *t* test).

First, western blot (WB) analysis showed the endogenous expression levels of CILP in MDA-MB-231, MDA-MB-468, and MCF-7 cell lines (Figure 4A). To demonstrate the role of CILP, we overexpressed CILP by transient transfection in MDA-MB-231 and MCF-7 cells (Figures 4B,C). *In vitro* colony formation and CCK-8 assays confirmed that CILP overexpression suppressed the proliferation of BC cells (Figures 4D,E). Wound healing and transwell assays showed that CILP overexpression reduced the migratory and invasive abilities of BC cells (Figures 4F,G). Vimentin is regarded as a key marker of the epithelial–mesenchymal transition (EMT) process. Furthermore, the WB result showed that the mesenchymal protein marker vimentin was significantly downregulated upon the CILP overexpression (Figure 4H). These results demonstrate that CILP inhibited BC proliferation, migration, and invasion *in vitro*.

### 3.5 CILP May Be a Putative Gene Associated With Breast Cancer Brain Metastases

**FIGURE 5:**
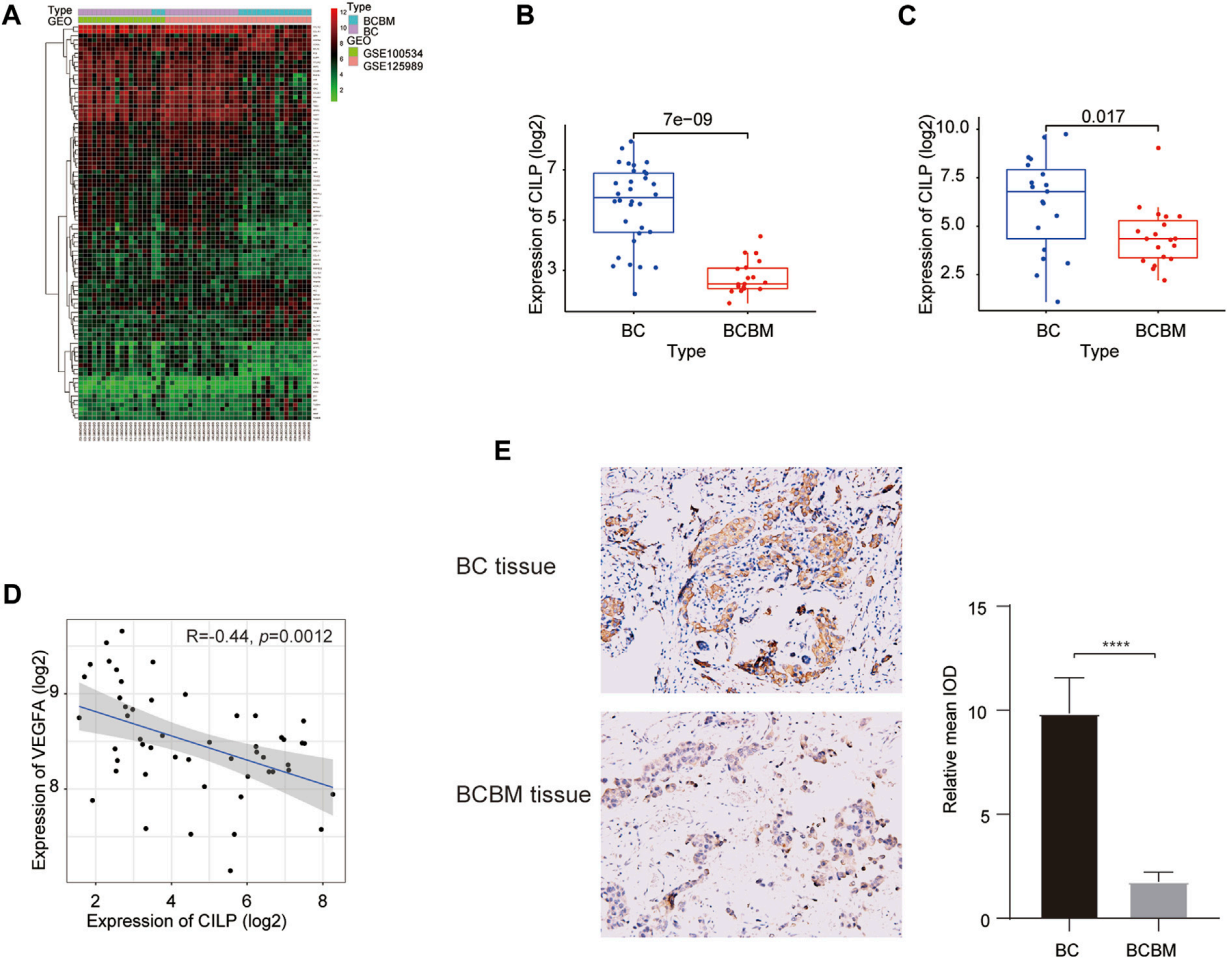
Differences in CILP expression between BCBM and BC. **(A)** Heatmap of 90 DEGs (28 up and 62 downregulated genes) in the merged datasets of GSE125989 and GSE100534. Green, black, and red represent a lower expression level, medium expression level, and higher expression difference among the genes, respectively. Type: Blue and purple represent BCBM and BC tissues, respectively. **(B,C)** Box plots showing CILP expression in BCBM and BC were analyzed using Wilcoxon rank-sum test. **(D)** Correlation between CILP and VEGFA mRNA expression levels (log2) was analyzed using the Spearman correlation analysis. **(E)** Representative immunohistochemistry staining images of CILP in BCBM and BC tissues (20×). Data **(A,B,D)**: GSE125989 and GSE100534; Data **(C)**: GSE43837; *p < 0.05, **p < 0.01, ***p < 0.001, and ****p < 0.0001.

We used the “limma” package to identify the differentially expressed genes (DEGs) between BCBM and BC in the merged GEO datasets. The heatmap showed that 90 DEGs were identified, of which 62 were downregulated and 28 were upregulated (adjusted *p* value < 0.05, |log2FC| ≥ 1) (Figure 5A). The CILP gene is a candidate hub gene for the yellow module (Figures 1G,J), which had the highest correlation with BCBM, and its expression was significantly downregulated in BCBM compared with that in BC (Figure 5B). In GSE43837, the CILP expression in BCBM was lower than that in BC (Figure 5C). It is well-known that CILP antagonizes transforming growth factor-beta (TGF-β) and insulin-like growth factor 1 (IGF1). The TGF-β signaling pathway is instrumental in the occurrence and development of cancer. Therefore, CILP was selected as the potential hub gene for further investigation. VEGFA overexpression contributes to unlimited tumor growth and vascularization in BC, especially TNBC (Linderholm et al., 2009; Srabovic et al., 2013). Moreover, VEGFA is involved in the process of BCBM (Chang et al., 2020). We found that CILP mRNA expression was moderately associated with VEGFA expression (*p* = 0.0012, *r* = −0.44; Figure 5D). We also performed immunohistochemistry to verify the CILP expression in BCBM and BC. The CILP expression was significantly downregulated in BCBM tissues compared with that in the BC tissues (Figure 5E). Overall, the abovementioned results demonstrate that CILP may be a putative gene affecting BCBM.

### 3.6 CILP Is Associated With the T-Cell Receptor Signaling Pathway

**FIGURE 6:**
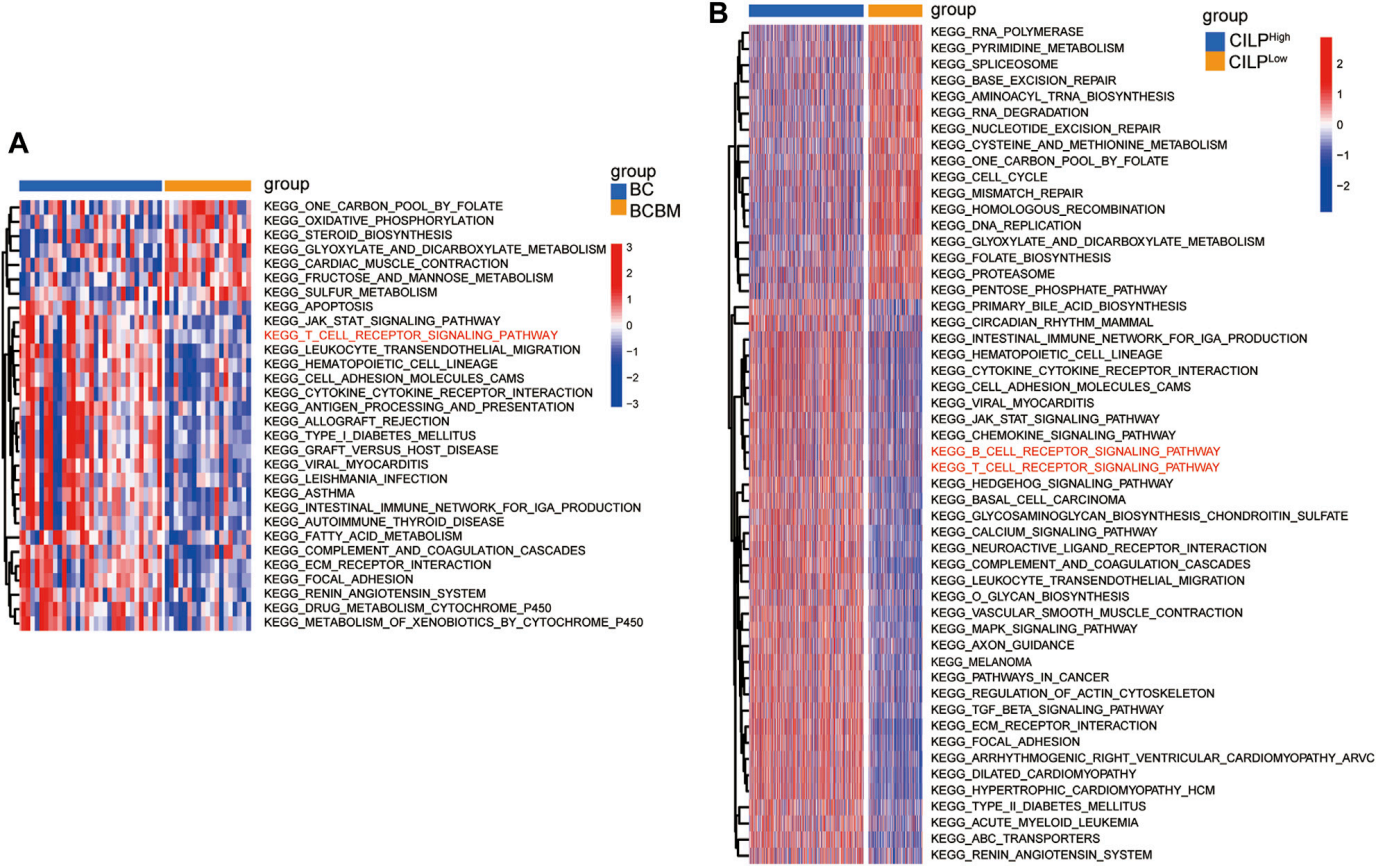
CILP is associated with the T-cell receptor signaling pathway analyzed using GSVA. **(A)** Heatmap for pathway activities was scored using GSVA between the BCBM and BC groups. **(B)** Differences in pathway activities between the high- and low-CILP expression groups in BC were analyzed using GSVA. **(A)** GSE125989 and GSE100534 cohort. **(B)** TCGA cohort.

To explore the biological processes of BCBM, GSVA was conducted using the merged datasets GSE100534 and GSE125989. The BCBM group was significantly associated with the immune-related biological processes, such as ECM–receptor interaction, focal adhesion, JAK-STAT signaling pathway, T-cell receptor signaling pathway, leukocyte transendothelial migration, antigen processing and presentation, and complement and coagulation cascades (Figure 6A). The previous results showed thatCILP expression was downregulated in BCBM compared with that in BC. Surprisingly, GSVA analysis using the TCGA datasets revealed that low expression of CILP was prominently related to the T- and B-cell receptor signaling pathways in BC (Figure 6B).

### 3.7 Breast Cancer Brain Metastases Displays Significantly Lower Immune Infiltration Than Breast Cancer

**FIGURE 7:**
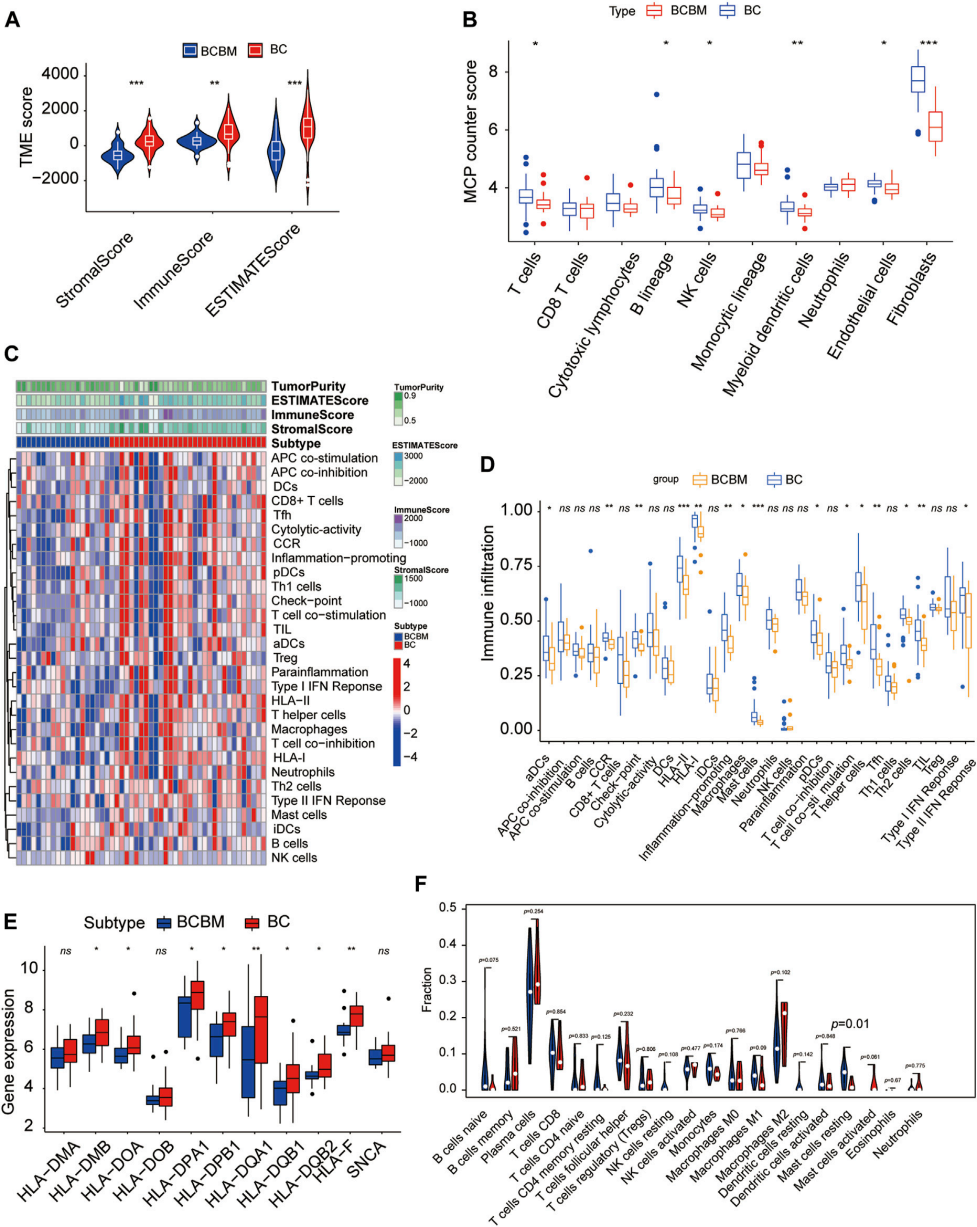
BCBM and BC show differences in immune infiltration. **(A)** Stromal score, immune score, and estimate score were calculated *via* the ESTIMATE method between the BCBM and BC groups. **(B)** MCP-counter analysis of the immune cell populations in BCBM (*n* = 19) and BC (*n* = 32) groups. **(C)** Heatmap using ssGSEA scores from the 29 immune components between the BCBM and BC groups. **(D)** Comparison of the 29 immune components between the BCBM and BC groups. **(E)** Expression of HLA-II molecules among BCBM and BC groups. **(F)** Violin plot shows the differential proportion of tumor-infiltrating immune cells between the BCBM and BC groups analyzed using CIBERSORT algorithm. Red and blue represent BCBM and BC, respectively. **(A,B,D–F)** Wilcoxon rank-sum test; **(A–F)** GSE125989 and GSE100534 cohort. *p < 0.05, **p < 0.01, and ***p < 0.001.

The degree of immune infiltration is highly correlated with tumor progression and prognosis. We used several algorithms to conduct the immune infiltration analysis on the BCBM and BC of the merged datasets GSE100534 and GSE125989, respectively. ESTIMATE was used to infer the degree of stromal and immune cell infiltration (Yoshihara et al., 2013). BCBM tissues showed a lower abundance of stromal and immune cells, and the immune scores decreased significantly in the BCBM tissues compared with those in the BC tissues (Figure 7A). The MCP-counter can quantify the infiltration population of 10 immune and stromal cells in tumors based on gene expression (Becht et al., 2016). The BCBM tissues showed significantly low levels of T cells (*p* < 0.05), B lineage (*p* < 0.05), natural killer cells (*p* < 0.05), myeloid dendritic cells (*p* < 0.01), endothelial cells (*p* < 0.05), and fibroblasts (*p* < 0.001). No difference was found in the levels of CD8^+^ T cells, cytotoxic lymphocytes, monocytic lineage, and neutrophils (Figure 7B). Furthermore, we evaluated the levels of 29 immune components, including immune pathways, immune factors, and immune cells, using the ssGSEA algorithm in 51 samples from the GEO datasets. The whole cohort was clustered into two clusters, BCBM and BC (Figure 7C), and the immune infiltration level is shown in Figure 7D. T-cell activation involves antigen-presenting cells (APCs) carrying the major histocompatibility complex (MHC) molecules that present antigens to the corresponding T-cell receptor on T cells. After the activation of CD4 T cells, type II interferon is secreted to play antitumor function. As shown in Figure 7D, CD4^+^ T-cell activation (including dendritic cell (DCs) ratio and expression of human leukocyte antigen (HLA)-II), CD4^+^ T-cell function (including T-cell co-stimulation, T helper cell level, and type II interferon response), and levels of macrophages, mast cells, HLA-I, T follicular helper cells (Tfh), type 2 T helper (Th2) cells, CCR, and checkpoint and tumor-infiltrating lymphocytes (TIL) were significantly lower in the BCBM group than those in the BC group. Further analysis showed that the molecular subtypes of HLA-II class was significantly lower in the BCBM tissues than in those with the BC tissues (Figure 7E). Subsequently, we used the CIBERSORT algorithm to calculate the constituent proportions of the 20 immune cells subtypes. Surprisingly, only the resting mast cells showed lower infiltration in the BCBM group (*p* = 0.01) (Figure 7F). Evidence indicated that the BCBM tissues displayed significantly lower immune infiltration than the BC tissues, especially the CD4^+^ T-cell activation and function.

### 3.8 CILP Was Associated With Immune Infiltration in Breast Cancer Brain Metastases

**FIGURE 8:**
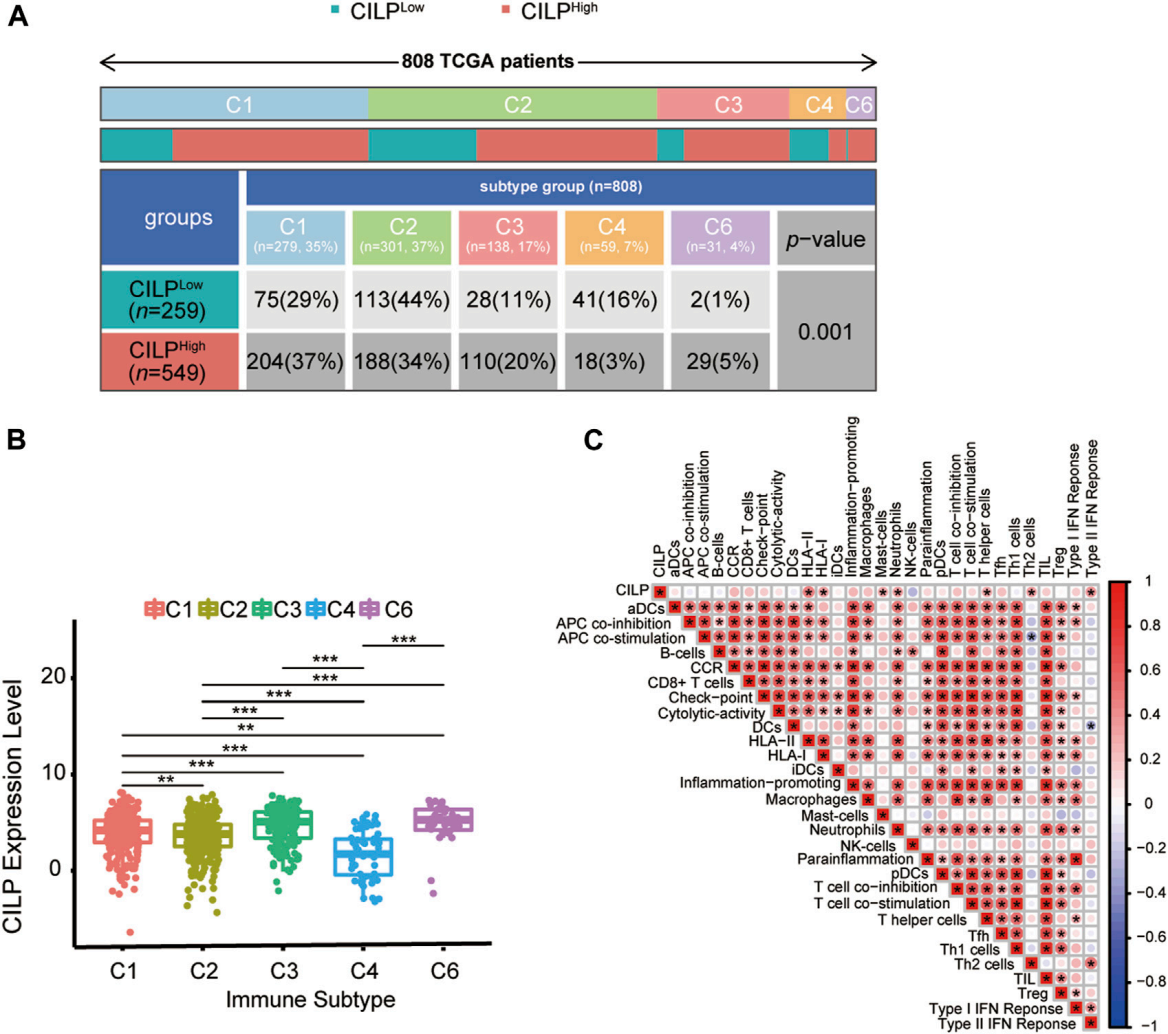
Expression of CILP correlates with immune infiltration level. **(A)** Differences in immune subtype between the high- and low-CILP expression groups in BC were analyzed using the Chi-square test. **(B)** Analysis of CILP mRNA expression in different immune subtypes of BC. **(C)** Correlation between CILP and the infiltrating immune components. The *p* value of the data **(B)** are calculated by Wilcoxon rank-sum test. **(C)** Spearman correlation analysis. **(A,B)** TCGA cohort; **(C)** GSE125989 and GSE100534 cohort *p < 0.05, **p < 0.01, and ***p < 0.001.

Recently, attention has focused on the importance of immunity in BC. Across cancer types, tumors have been identified in six immune subtypes: wound healing (C1), IFN-γ dominant (C2), inflammatory (C3), lymphocyte depleted (C4), immunologically quiet (C5), and TGF-β dominant (C6), characterized by differences in molecular and immune features, and these subtypes are related to the survival of patients (Thorsson et al., 2018). Chi-square analysis revealed that the CILP expression was significantly associated with the immune subtypes in BC (Figure 8A). However, there was no patient with immunologically quiet (C5) subtype BC. Because C5 subtype, which showed the lowest lymphocyte, is dominated by the low-grade gliomas (Thorsson et al., 2018). CILP was significantly downregulated in C4 subtype, which have a lower ratio of Th1:Th2, compared with the other immune subtypes BC (Figure 8B). Overall, the results showed that low CILP expression was related to depleted lymphocyte.

As shown in Section 3.6, CILP was involved in the T-cell pathway in BC. Hence, it is necessary to explore the immune effects of CILP in BCBM. The results of the ssGSEA showed that CILP expression had a significantly positive correlation with the HLA-II, T helper cells (CD4^+^ T cells), and Type II IFN response in the BC and BCBM groups of merged GEO datasets (Figure 8C). In short, CILP was associated with the activation and function of CD4^+^ T cells in BCBM.

## 4 Discussion

In our study, we found that CILP was the hub gene, which was significantly associated with BCBM. CILP, cartilage intermediate layer protein 1, plays a crucial role in the lumbar disc disease *via* negative regulation of TGF-β signaling (Seki et al., 2005). Elevated TGF-β levels are associated with poor outcomes in several different tumor types (Massague, 2008; Lin and Zhao, 2015). Furthermore, TGF-β promotes tumor progression by influencing angiogenesis, inducing EMT, and immune suppression (Lebrun, 2012; Principe et al., 2014). This may support the hypothesis that CILP could contribute to the occurrence of BCBM through the TGF-β pathway. CILP has also been shown to inhibit the proliferation and migration of BC cells *in vitro*, but whether CILP is involved in BCBM through the TGF-β pathway requires further study. Moreover, it is reasonable to assume that CILP may represent a new biomarker, indicative of BCBM.

In the context of the highly immunosuppressive microenvironment of the BM, it is essential to activate the antitumor T-cell responses effectively (Quail and Joyce, 2017). Our study also identified immunosuppressive microenvironment in BCBM compared with that in BC, especially CD4^+^ T-cell activation and function. The low expression of CILP is closely related to BCBM. Interestingly, we also found that a significantly positive correlation between the CILP expression and HLA-II, T helper cells (CD4^+^ T cells), and type II IFN response in BCBM. These results suggested that CILP, which may inhibit BCBM, is a potential regulatory node for CD4^+^ T-cell activation and function. The CD4^+^ T cells play an antitumor immunomodulatory role by secreting interferon-gamma (IFN-γ). Previous studies have suggested that IFN-γ induces an effective antitumor immune response by enhancing tumor antigen presentation due to the increased expression of proteins, such as MHC molecules, involved in antigen presentation (Platanias, 2005). Our study suggests that a low expression of CILP could induce occurrence of BCBM partly due to the reduced antitumor effects of the CD4^+^ T cells. TGF-β plays a biological role in promoting immune escape in advanced tumor. It has been reported that TGF-β directly targets CD4^+^ T-cell metabolism in metastatic tumor effusions, resulting in reduced IFN-γ production, and thereby inhibiting the CD4^+^ T-cell function (Dimeloe et al., 2019). Therefore, it is suggested that reducing the TGF-β-induced metabolic impairment of CD4^+^ T cells is an effective antitumor strategy. Whether CILP can reduce T-cell metabolic disorder and thus inhibit the occurrence of BCBM by inhibiting TGF-β function remain to be confirmed by further studies.

However, our study has several limitations. The GEO database contains limited datasets on BC and BCBM with clinical information, thus there are not enough external datasets to validate our findings. Therefore, we have started to establish the BC and BCBM clinical datasets and performed preliminary immunohistochemical analysis of CILP in BC and BCBM. Research on the relationship between CILP and clinicopathological features and prognosis is in progress. The relationship between CILP and BCBM could not be verified in animal models due to the difficulty in modeling BM (a preliminary attempt was made to establish a model by caudal vein injection and left ventricular injection of the BC cells, both of which were unsuccessful and are still being explored). Regarding mechanism speculation, only the correlation between CILP and the tumor immune microenvironment was identified, but it fails to penetrate specific pathways or targets, which requires further study.

In summary, our study indicated that CILP was associated with immune infiltration, and it may be a putative gene involved in BCBM. CILP offers new insights into the pathogenesis of BCBM, which will facilitate the development of novel targets for the patients with BCBM.

## Data Availability

The original contributions presented in the study are included in the article/Supplementary Material, further inquiries can be directed to the corresponding authors.
